# Data on the use of virgin coconut oil and bioethanol produced from sugar palm sap as raw materials for biodiesel synthesis

**DOI:** 10.1016/j.dib.2020.105199

**Published:** 2020-01-25

**Authors:** Sanusi Gugule, Feti Fatimah, Chaleb Paul Maanari, Trina Ekawati Tallei

**Affiliations:** a Department of Chemistry, Faculty of Mathematics and Natural Sciences, Manado State University, Tondano, North Sulawesi, Indonesia; b Department of Chemistry, Faculty of Mathematics and Natural Sciences, Sam Ratulangi University, Manado, North Sulawesi, Indonesia; c Department of Biology, Faculty of Mathematics and Natural Sciences, Sam Ratulangi University, Manado, North Sulawesi, Indonesia

**Keywords:** Biodiesel, Bioethanol, Sugar palm, Sap, Transesterification, Virgin coconut oil

## Abstract

These data describe the use of virgin oil from coconuts (*Cocos nucifera*) and bioethanol from the sap of sugar palms (*Arenga pinnata*) as raw materials for biodiesel synthesis. Virgin coconut oil (VCO) was produced using mechanical techniques and was fermented without heating, while bioethanol was obtained from the distillation/redistillation of sugar palm sap (SPS), which was fermented spontaneously. Biodiesel was obtained by refluxing VCO and SPS bioethanol with a potassium hydroxide (KOH) transesterification catalyst for 3 hours. The results of the reaction were tested by determining the physical and chemical properties as well as by identifying the main components of biodiesel by spectroscopy. The gas chromatography-mass spectrometry (GC-MS) chromatogram data and spectrum identification results show that ethanol from the distillation/redistillation of spontaneously fermented SPS can be used as a reagent in biodiesel synthesis.

**Table:** Specifications Table

Subject area	Sustainable energy
More specific subject area	Renewable energy
Type of data	Tables and figures
How data were acquired	Infrared spectrophotometry, gas chromatography-mass spectrometry (GC-MS), and ^1^H NMR spectrometry
Data format	Raw and analyzed
Experimental factors	Bioethanol was obtained by distillation and redistillation of fermented sugar palm sap. Virgin coconut oil was produced from coconut flesh with mechanical techniques without heating and biodiesel was synthesized by transesterification.
Experimental features	Biodiesel was produced by a transesterification process.
Data source location	North Sulawesi, Indonesia
Data accessibility	Data are available within this article

**Table:** 

**Value of the Data** • These data can be used as a reference for synthesizing biodiesel from sugar palm sap (SPS) bioethanol and virgin coconut oil (VCO) with a potassium hydroxide catalyst. • These data are very useful for the community and stakeholders for developing and increasing the economic value of SPS and VCO. In addition, these data are very useful to researchers as preliminary information for the development of bioethanol/biodiesel synthesis with the availability of cheap and abundant natural raw materials in North Sulawesi, Indonesia. • The method for obtaining data in this study is very clear, has a high repeatability, and is supported by accurate spectroscopic and chromatographic data, and it can be used as a basis for further research development.

## 1 Data

### 1.1 Characteristics of the VCO

From 1 kg of freshly grated coconut mixed in 1.5 L of coconut water, 219 mL (or 202.77 g, ρ = 0.9259 g/mL, 20.28%) of virgin coconut oil (VCO) was collected. Using the results of the VCO quality testing, the following data were obtained: an Iod number of 9, an acid number of 0.16, a peroxide number of 0.28, a moisture content of 0.45%, and a density of 0.9259 g/mL.

### 1.2 Fatty acid profile of the VCO

The fatty acid profile of the VCO was analyzed by gas chromatography-mass spectrometry (GC-MS) using an internal standard of margaric acid. Interpretations of the chromatogram data are presented in Table 1, which shows the average percentages of saturated and unsaturated fatty acids.

**Table 1:** Average percentages of fatty acids in VCO, based on GC-MS analysis.

Types of Fatty Acids	Average Percentage of Fatty Acid (%)
**Saturated Fatty Acids**	
Caprylic acid (C8:0)	6.19
Capric acid (C10:0)	5.86
Lauric acid (C12:0)	49.53
Myristic acid (C14:0)	19.27
Palmitic acid (C16:0)	9.51
Stearic acid (C18:0)	2.96
**Total saturated fatty acids**	**93.32**
**Unsaturated Fatty Acids**	
Oleic acid (C18:1,n-9)	5.60
Linoleic acid (C18:2,n-6)	0.95
α-Linoleic acid (C18:3,n-3)	0.04
**Total unsaturated fatty acids**	**6.59**
**Total fatty acids**	**100.00**

### 1.3 Characteristics of bioethanol derived from sugar palm sap

The distilled sugar palm sap (SPS) A (from the first distillation) produced by the local community had a bioethanol level of 44.45% (ρ = 0.9395 g/mL), and the distilled SPS B (from the second distillation) had a bioethanol level of 80.05% (ρ = 0.8571 g/mL). The data from the distillation/redistillation of bioethanol derived from distilled SPS C (the third distillation) are shown in Table 2. The average bioethanol level obtained from SPS C was 89.96%.

**Table 2:** Data for the specific gravity of SPS bioethanol C at 25 °C.

Repetition of the distillation process	Specific gravity	SPS bioethanol C Level (%)
I	0.8370	90.02
II	0.8372	89.96
III	0.8375	89.87
**Average**	**0.8372**	**89.96**

**Fig. 1:**
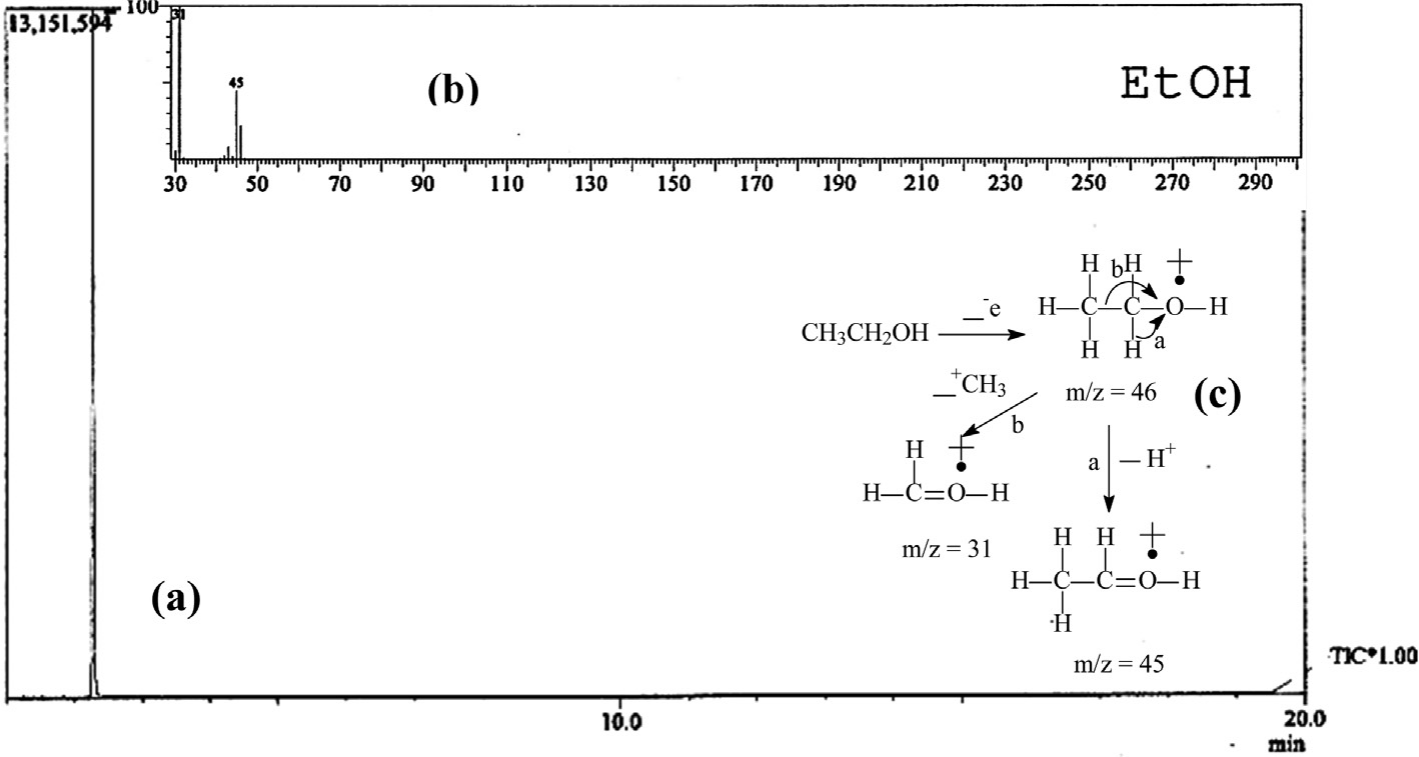
(a) SPS bioethanol chromatogram, (b) mass spectrum of SPS bioethanol, and (c) SPS bioethanol fragmentation pattern.

The SPS bioethanol chromatogram and mass spectrum presented in Fig. 1 (a) and (b), respectively, show a single peak. This result means that the SPS bioethanol concentration was 100%. The SPS bioethanol fragmentation pattern is presented in Fig. 1(c).

The infrared spectrum presented in Table 3 shows the characteristics of the functional groups of the SPS bioethanol structure. A wide absorption peak at 3200-3600 cm^−1^ corresponds to the absorption of the hydroxyl alcohol group (-OH). These data are supported by the absorption of SPS bioethanol C-O groups at 1049-1273 cm^−1^. Likewise, absorption of an alkyl group (C-H) is indicated by the peak at 2893-2978 cm^−1^, which is confirmed by the absorption of the methylene group (-CH_2_-) at 1450 cm^−1^ and of the methyl group (-CH_3_) at 1327 cm^−1^.

**Table 3:** Infrared absorption data of SPS bioethanol.

υ (cm^−1^)	Absorption
3200-3600 (stretching)	-OH (hydroxyl alcohol group)
2893-2978 (stretching)	C-H (alkyl group)
1450 (bending)	-CH_2_- (methylene group)
1327 (bending)	-CH_3_ (methyl group)
1049–1273	C-O (alcohol group)

The ^1^H NMR spectrum of SPS bioethanol (Table 4) indicates the existence of a triplet peak (t) at chemical shift values (δ) of 0.9–1.3 ppm. This peak shows the presence of a methyl group (-CH_3_) next to the two hydrogen atoms of the methylene group (-CH_2_-). There is also a quartet peak (q) at chemical shift values (δ) of 3.2–3.7 ppm. This peak shows the presence of a methylene group (-CH_2_-) next to the three hydrogen atoms of the methyl group (-CH_3_). Likewise, there is a singlet peak (s) at a chemical shift value of 4.3 ppm. This peak shows the absorption of the one hydrogen atom from the hydroxyl group (-OH). The chromatogram and spectrum data show the structure of SPS bioethanol compounds. This SPS bioethanol was used as a reagent in biodiesel synthesis.

**Table 4:** Interpretation of ^1^H NMR of SPS bioethanol.

δ (ppm)	Multiplicity	H Atom Position
0.9–1.3	t	CH_3_ (d)
3.2–3.7	q	CH_2_ (c)
4.3	s	OH (b)

### 1.4 Biodiesel characteristics

The transesterification reaction yielded 164.04 mL (144.16 g) biodiesel (ethyl ester). The biodiesel was clear in appearance and free of visible water and had an average yield of 78.35%. Table 5 shows that the heat capacity of the biodiesel was 9763.820 cal/mol and that the specific gravity of the biodiesel at 40 °C was 878.9 kg/m^3^. This value is still within the SNI range, even though it is at the maximum limit. From Table 5, it can also be seen that the biodiesel refractive index was 1.451. The data also showed that the viscosity of biodiesel was 5.23 mm^2^/s. This value is still in the range of standards set by the SNI.

**Table 5:** Characteristics of biodiesel resulting from the synthesis of SPS bioethanol and VCO using KOH.

No	Tested Parameters	Unit	Test Results	SNI Specifications^a^ 7182:2015		Test Method
			Biodiesel	Min	Max	
1	Specific gravity at 40 °C	kg/m^3^	878.9	850	890	ASTM D 4052
2	Viscosity at 40 °C	mm^2^/s	5.23	2.3	6.0	ASTM D 445
3	Cetane number	–	48.5	51	–	ASTM D 4613
4	Water and sediment	%vol	0	–	–	ASTM D 2709
5	Heat capacity	cal/mol	9763.820	–	–	–
6	Refractive index	–	1.451	–	–	–

a SNI (*Standard Nasional Indonesia* or Indonesian National Standard).

The biodiesel cetane number was 48.5, which is still less than the minimum SNI standard value of 51. High cetane numbers are good for increasing the combustion ability. The cetane number is indicative of the time delay associated with the ignition of diesel fuel during injection into the combustion chamber. The cetane number is influenced by several factors, such as the presence of unsaturated fat components, the number of double bonds, and the length of the bonds. The data from the infrared spectrum of the biodiesel are presented in Table 6.

**Table 6:** Infrared spectrum of SPS biodiesel.

Υ (cm^−1^)	Absorption
2854, 2924 (stretching)	C-H (alkyl group)
1743 (bending)	-CO- (carbonyl group)
1458 (bending)	-CH_2_-(methylene group)
1373 (bending)	-CH_3_ (methyl group)
1165, 1111	C-O (ester group)

The infrared spectrum data presented in Table 6 show the characteristics of the functional groups in the structure of ethyl ester (biodiesel). The absorption of the alkyl group (C-H) at 2854 and 2924 cm^−1^ was confirmed by the absorption of the methylene group (-CH_2_-) at 1458 cm^−1^ and of the methyl group (-CH_3_) at 1373 cm^−1^. There is sharp absorption at 1743, which shows the absorption of the carbonyl groups from the esters. These data are supported by the absorption of C-O ester groups at 1111 and 1165 cm^−1^. Thus, the identified compounds contain carbonyl groups, alkyl groups (methyl and methylene), and the C-O groups of esters. Furthermore, a chromatogram with nine peaks was obtained from the GC-MS data for the identification of biodiesel components. The biodiesel chromatogram data are presented in Table 7.

**Table 7:** Main components of biodiesel.

Peak	Area (%)	Name of components
2	6.43	Ethyl octanoate (ethyl caprylate)
3	5.75	Ethyl decanoate (ethyl caprate)
4	51.14	Ethyl dodecanoate (ethyl laurate)
5	18.40	Ethyl tetradecanoate (ethyl myristate)
6	8.55	Ethyl hexadecanoate (ethyl palmitate)
9	5.48	Ethyl octadecanoate (ethyl stearate)

**Fig. 2:**
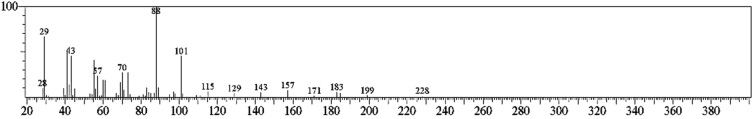
Mass spectrum of component 4.

**Fig. 3:**
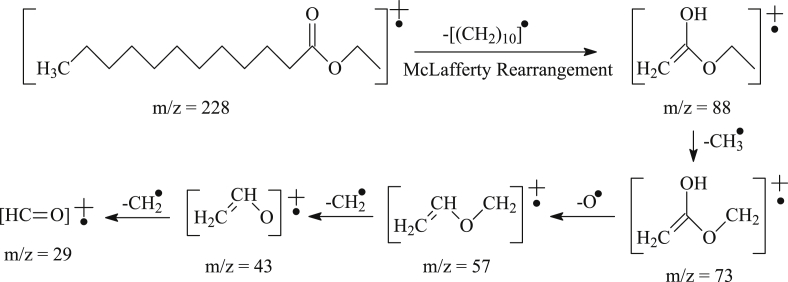
Fragmentation pattern of ethyl laurate.

From Table 7, it is observed that there were six predominant biodiesel components, including ethyl caprylate, ethyl caprate, ethyl laurate, ethyl myristate, ethyl palmitate, and ethyl stearate. Because the ethyl ester fragmentation pattern is the same as the fragmentation pattern of other fatty acids, we next present the fragmentation pattern of the component that has the highest concentration and is the most dominant component of biodiesel, namely, ethyl laurate at 51.14% (peak 4, with a retention time of 30.952). The mass spectrum of peak 4 shows similarity to the mass spectra of ethyl lauric compounds. The mass spectrum image of component 4 and the ethyl laurate fragmentation pattern (some of which followed McLafferty's rearrangement [1]) are presented in Fig. 2, Fig. 3, respectively. The infrared spectrophotometry and GC-MS data show that the distilled/redistilled SPS bioethanol reached 89–90% and could be used as a reagent in biodiesel synthesis. The data show that VCO and naturally fermented SPS bioethanol can be used as raw materials for the production of biodiesel.

## 2 Experimental design, materials, and methods

### 2.1 Separation and purification of virgin coconut oil

Old coconuts were peeled, and the flesh was removed from the shells. The brown-colored part of the outer layer of flesh was removed using a knife, and the coconut was shredded using a grater. As much as 1000 g of grated coconut was added to 1.5 L of fresh coconut water, and the mixture was squeezed to obtain coconut milk. The coconut milk was left to stand for approximately 30 minutes until two layers were formed. The coconut cream layer was removed and stirred for 1 hour using a mixer at full speed, and the mixture was left to stand for 10 hours until it was separated into three layers: the top layer (oil-rich), middle-layer or skim (protein-rich), and bottom layer (sediment). After the oil-rich layer (*blondo*) was separated from the rest of the layers, it was left to stand and was filtered with 400 mesh filter paper. The produced oil was weighed, and its peroxide number, saponification number, acid number, specific gravity, and lipid profile were determined by GC-MS using a margaric acid internal standard. The fatty acid levels in the coconut oil were calculated using the following formula [2]: (calculated fatty acids)/(total area − solvent area − internal standard area) × 100.

### 2.2 SPS bioethanol separation and purification

The density of 10 mL distilled SPS A produced by the local community was determined to obtain data on SPS bioethanol and pH levels. Afterward, 500 mL of the distillate was redistilled using a distillation apparatus with a 75 cm long Vigreux column, and the distillation temperature was maintained at 71–73 °C to produce distilled SPS B. The distillate was then redistilled again under the same conditions to produce distilled SPS C. The SPS bioethanol level and pH of distillates B and C were measured using a pycnometer and were then compared with similar data based on the literature [2]. Further distillation was carried out to obtain distillate that was estimated to have maximum bioethanol content. The bioethanol level and pH of this redistilled bioethanol (C) were tested. The structure identification was carried out using infrared spectrophotometer (IR Prestige-21 SHIMADZU), gas chromatography-mass spectrometer (GC-MS-QP2010S SHIMADZU), and ^1^H NMR spectrometer (HITACHI FT-NMR-R-1900).

### 2.3 Biodiesel synthesis from VCO and SPS bioethanol

As much as 200 mL of VCO (184 g) was heated at a temperature of 50 °C in a three-neck flask equipped with a condenser and then combined with 2% KOH, which was made by dissolving 3.68 g KOH in 250.50 mL of SPS bioethanol. The mixture was refluxed stirred at 71–75 °C for 3 hours and left to stand in a separating funnel for 24 hours. Next, 200 mL of distilled water was added, and the mixture was left to stand for 24 hours. The top layer was taken out and washed with warm water until it was clear and heated to a temperature of 105 °C. The resulting biodiesel was then ready to be analyzed.

### 2.4 Characteristic assay of biodiesel

The characteristic assay of the biodiesel produced from the transesterification reaction included determination of the specific gravity, viscosity (Ostwald), cetane number, water and sediment contents, heat capacity, and refractive index (Abbe-type refractometer). Spectroscopic structure identification was carried out using infrared spectrophotometer (IR Prestige-21 SHIMADZU) and gas chromatography-mass spectrometer (GC-MS-QP2010S SHIMADZU).
